# Cervical Carcinoma: Evaluation Using Diffusion MRI With a Fractional Order Calculus Model and its Correlation With Histopathologic Findings

**DOI:** 10.3389/fonc.2022.851677

**Published:** 2022-04-05

**Authors:** Xian Shao, Li An, Hui Liu, Hui Feng, Liyun Zheng, Yongming Dai, Bin Yu, Jin Zhang

**Affiliations:** ^1^Department of Anesthesiology, The Fourth Hospital of Shijiazhuang, Shijiazhuang, China; ^2^Department of Radiology, The Fourth Hospital of Hebei Medical University, Shijiazhuang, China; ^3^MR Collaboration, Central Research Institute, United Imaging Healthcare, Shanghai, China; ^4^Department of Emergency, The Fourth Hospital of Hebei Medical University, Shijiazhuang, China

**Keywords:** magnetic resonance imaging, diffusion-weighted imaging, cervical carcinoma, cervical squamous cell carcinoma, cervical adenocarcinoma

## Abstract

**Objective:**

The objective of the study is to investigate the feasibility of using the fractional order calculus (FROC) model to reflect tumor subtypes and histological grades of cervical carcinoma.

**Methods:**

Sixty patients with untreated cervical carcinoma underwent multi-b-value diffusion-weighted imaging (DWI) at 3.0T magnetic resonance imaging (MRI). The mono-exponential and the FROC models were fitted. The differences in the histological subtypes and grades were evaluated by the Mann–Whitney U test. Receiver operating characteristic (ROC) analyses were performed to assess the diagnostic performance and to determine the best predictor for both univariate analysis and multivariate analysis. Differences between ROC curves were tested using the Hanley and McNeil test, while the sensitivity, specificity, and accuracy were compared using the McNemar test. *P*-value <0.05 was considered as significant difference. The Bonferroni corrections were applied to reduce problems associated with multiple comparisons.

**Results:**

Only the parameter β, derived from the FROC model could differentiate cervical carcinoma subtypes (*P* = 0.03) and the squamous cell carcinoma (SCC) lesions exhibited significantly lower β than that in the adenocarcinoma (ACA) lesions. All the individual parameters, namely, ADC, β, *D*, and μ derived from the FROC model, could differentiate low-grade cervical carcinomas from high-grade ones (*P* = 0.022, 0.009, 0.004, and 0.015, respectively). The combination of all the FROC parameters showed the best overall performance, providing the highest sensitivity (81.2%) and AUC (0.829).

**Conclusion:**

The parameters derived from the FROC model were able to differentiate the subtypes and grades of cervical carcinoma.

## Introduction

Cervical cancer is the fourth most common cancer in women worldwide (1). In developing countries, probably due to the poor access to the screening programs, most of the new cases present advanced stages and remain poor prognosis. Both histological subtypes and grades are the most common prognostic factors of cervical cancer. The two main histological subtypes, squamous cell carcinoma (SCC) and adenocarcinoma (ACA), account for approximately 70 and 25% of all cervical cancers, respectively (1). According to a previous study, the different cell types probably have different patterns of failure and survival (2). The aggressiveness of tumors represented by histological grade can also provide critical information for selecting treatment plans. Therefore, the clear insights into tumor subtypes and histological grades are essential in cervical carcinoma diagnosis and management. Despite biopsy being currently performed for the assessment of cervical carcinoma and is considered as a gold standard for patients presenting with advantage stages, it is an invasive procedure associated with certain risks of bleeding and infection, and sampling bias may occur especially for larger tumors (3, 4).

Magnetic resonance imaging (MRI) emerged as a powerful noninvasive diagnostic tool in oncology. Among MRI techniques, diffusion-weighted imaging (DWI) probes tissue microenvironment based on its sensitivity to water molecular diffusion that can be quantified using apparent diffusion coefficient (ADC) derived from the Gaussian diffusion model. Previous studies reported the potential use of ADC to evaluate cervical carcinoma subtypes and grades (5–7). However, this conventional mono-exponential diffusion model lacks specific parameters to reflect tumor microstructures, which are essential for assessing tumor subtypes and grades. According to the previous studies, the differentiation of cervical carcinoma histological grades based on ADC alone was reported to be difficult as the overlapped ADC values among different histological grades would act as a confounder (8, 9). Similarly, some overlap in ADC values were found between SCC and ACA (6).

Recently, the fractional order calculus (FROC) model was suggested to evaluate the microstructural and heterogeneity changes in tumor tissues. The FROC model provides a new set of parameters, including an anomalous diffusion coefficient *D*, an intravoxel diffusion heterogeneity parameter β, and a spatial parameter *μ* (10). Collectively, these parameters offer a multi-faceted characterization of cancerous tissues and can be used as a new class of biomarker in tumor diagnosis (11–14). Prior research proved that the FROC model could better reflect the complexity and heterogeneity of tissue microstructure than conventional diffusion model in gastric adenocarcinoma (15) and prostate lesions (16).

This study aimed to investigate whether the parameters derived from the FROC model can be used for imaging-based assessment of histological subtypes and grades of cervical carcinoma. Furthermore, the diagnostic performance results of ADC and the FROC parameters were compared to find the best predictor.

## Materials and Methods

### Study Population

This prospective study was approved by our institutional review board and written informed consents were obtained from all participants. The inclusion criteria were as follows: (1) no previous treatment for cervical carcinoma prior to the MR examination; (2) no contraindications to the MR examination; (3) clinically and radiologically suspected cervical carcinoma patients. Exclusion criteria were as follows: (1) poor image quality; (2) rare histological subtypes; (3) lack of subtype classification and/or grade information through pathological evaluation. With these criteria, one patient with poor image quality, two patients with adenosquamous carcinoma, and two patients without pathological evaluation were excluded. Finally, between January 2021 to November 2021, sixty patients (mean age, 53.0 years ± 10.1 [standard deviation]) were enrolled.

All cases involved in this study were confirmed to have cervical carcinoma by the biopsy, which were further analyzed and reconfirmed by an experienced pathologist specialized in gynecological malignancies. Histological subtypes were separated into an SCC group and an ACA group. Besides, all the cases were classified into a high-grade group (poorly differentiated tumor) and a low-grade group (well- or moderately differentiated tumor).

### MR Imaging

All the MRI examinations were performed on a 3.0T MR scanner (uMR 780, United Imaging Healthcare, Shanghai, China) with a commercial 12-channel body phased array coil. MR sequences included: 1) axial T1-weighted (T1W) fast spin echo (FSE) sequence; 2) axial T2-weighted (T2W) FSE sequence; 3) coronal fat-suppressed T2W FSE sequence; 4) sagittal T2W FSE sequence; and 5) diffusion-weighted imaging with a series of b-value 0, 20, 40, 80, 160, 200, 500, 1,000, and 2,000 s/mm^2^. **Table 1** presents all the detailed protocols.

**Table 1:** MRI protocols.

Parameters	Sequences				
	Axial T1W FSE	Axial T2W FSE	Coronal fat-suppressed T2W FSE	Sagittal T2W FSE	EPI-DWI
**TR (ms)**	586	2,268	4,139	2,103	6,152
**TE (ms)**	11.38	98.28	79.52	71.88	82.7
**Flip angle (°)**	130	105	105	105	90
**FOV (cm)**	22 × 22	22 × 22	22 × 22	22 × 22	30 × 26
**Matrix**	432 × 432	432 × 432	456 × 456	456 × 456	256 × 222
**Slice Thickness (mm)**	4	4	4	4	5
**Intersection gap (mm)**	0	0	0	0	0
**Bandwidth (Hz/pixel)**	200	220	200	180	2,120
**Number of slices**	30	30	23	23	25
**b-value (s/mm^2^)**	**/**	/	/	/	0, 20, 40, 80, 160, 200, 500, 1,000, 2,000

T1W, T1-weighted; T2W, T2-weighted; FSE, fast spin echo; EPI, echo-planar imaging; DWI, diffusion weighted imaging; TR, repetition time; TE, echo time; FOV, field of view.

### Image Analysis

The conventional mono-exponential diffusion model was applied to estimate ADC based on the images with two b-values, 0 and 1,000 s/mm^2^.

The FROC model was set up following the equation (17, 18)


(1) $$S=S_{0}\exp[-D\mu^{2(\beta -1)}{\left(\gamma G_{d}\delta \right)}^{2\beta }(\Delta -\frac{2\beta -1}{2\beta +1}\delta )]$$

where *S_0_* is the signal intensity without diffusion weighting, *G_d_* is the diffusion gradient amplitude, *D* is the anomalous diffusion coefficient, β is the intravoxel diffusion heterogeneity parameter, and μ is the spatial parameter.

Two experienced radiologists with 8 and 19 years of experience in gynecological imaging delineated the volumes of the interest (VOIs) using a 3D slicer (19). The VOIs were placed on the solid regions of tumors to avoid confounding effects caused by other tissue compositions, such as necrosis, mucinous lake, and calcification. The VOIs were first determined on the diffusion-weighted images with b = 0, and then propagated to the corresponding *D*, β, μ, and ADC map. The mean values of *D*, β, μ, and ADC were recorded.

### Statistics

Statistical analyses were performed with the SPSS software (Version 26, SPSS Inc., Chicago, IL, USA) and MedCalc (Version 20; MedCalc Software, Ostend, Belgium). The Mann–Whitney U tests were utilized to determine the statistical significance in mean parametric differences between the cervical carcinoma subtypes and differentiation grades. With the histopathological results as a gold standard, the receiver operating characteristic (ROC) analyses were performed on individual FROC parameters and ADC for the differentiation between a) SCC and ACA, and b) high-grade tumor and low-grade tumor. Then, the significant predictors were selected. Logistic regression analysis was performed to determine the optimal linear combination of these significant predictors in a model. The Youden index was exploited to determine the cutoff value for both univariate analysis and multivariate analysis, along with the area under the curve (AUC), sensitivity, specificity, and accuracy. Differences between ROC curves were tested using the Hanley and McNeil test (20), while the sensitivity, specificity, and accuracy were compared using the McNemar test. *P*-value <0.05 was considered as statistically significant. Bonferroni corrections were applied to reduce problems associated with multiple comparisons.

## Results

Patient demographics and tumor characteristics are given for all patients in **Table 2**. Of the 60 patients, 47 (78.3%) were SCC patients and 13 (21.7%) were ACA patients. Besides, there were 28 (46.7%) patients with low-grade tumors, while 32 (53.3%) patients with high-grade ones.

**Table 2:** Summary of the demographic and clinical features of the patients.

Variables	Patients
**Number (n)**	60
**Age, y (mean ± SD)**	53.0 ± 10.1
**Histological Subtypes, n (%)**	
** SCC**	47 (78.3%)
** ACA**	13 (21.7%)
**Tumor grade, n (%)**	
** Well-differentiated**	15 (25.0%)
** Moderately-differentiated**	13 (21.7%)
** Poorly differentiated**	32 (53.3%)

SD, standard deviation; SCC, cervical squamous cell carcinoma; ACA, cervical adenocarcinoma.

### Comparative Analysis of ADC and the FROC Parameters in Cervical Carcinoma Subtypes

**Figure 1:**
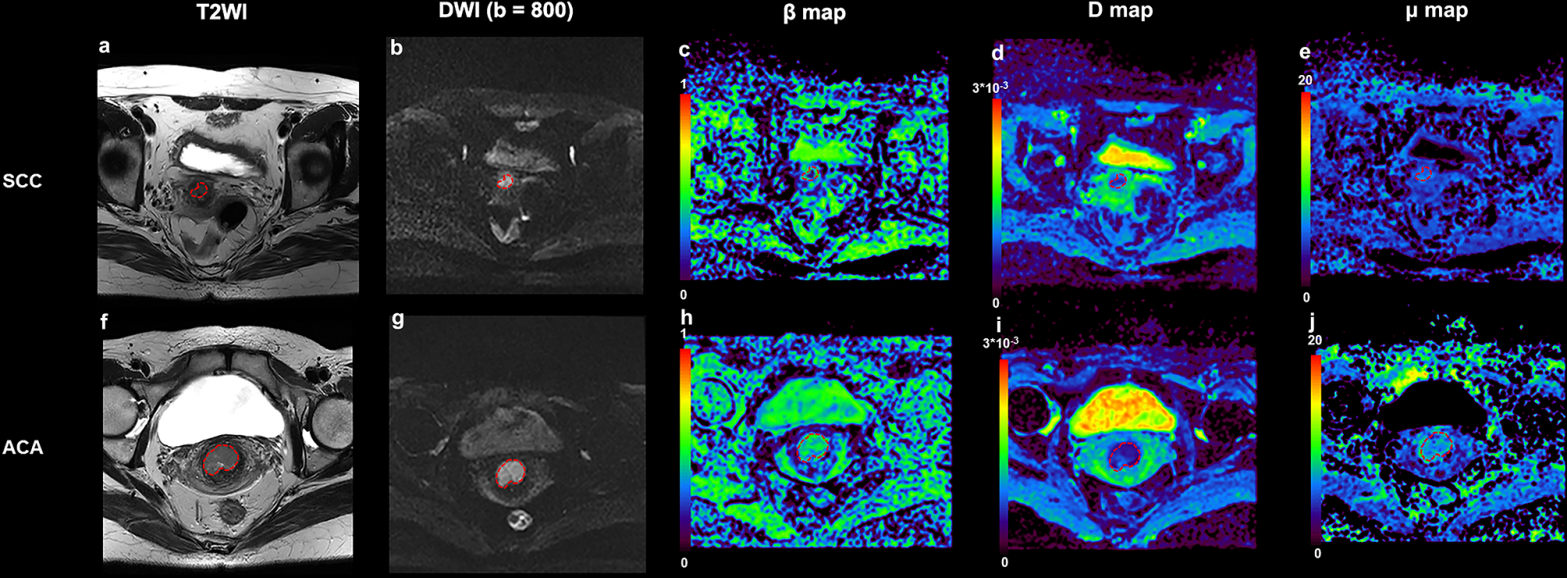
An example of SCC from a 70-year-old patient and ACA from a 56-year-old patient. **(A)** Transverse T2 image for patient with SCC; **(B)** DWI image (b = 800 s/mm^2^) for patient with SCC; **(C)** β map for patient with SCC; **(D)** *D* map for patient with SCC; **(E)** μ map for patient with SCC; **(F)** Transverse T2 image for patient with ACA; **(G)** DWI image (b = 800 s/mm^2^) for patient with ACA; **(H)** β map for patient with ACA; **(I)** *D* map for patient with SCC; **(J)** μ map for patient with SCC. SCC, cervical squamous cell carcinoma; ACA, cervical adenocarcinoma; DWI, diffusion-weighted imaging.

**Figure 2:**
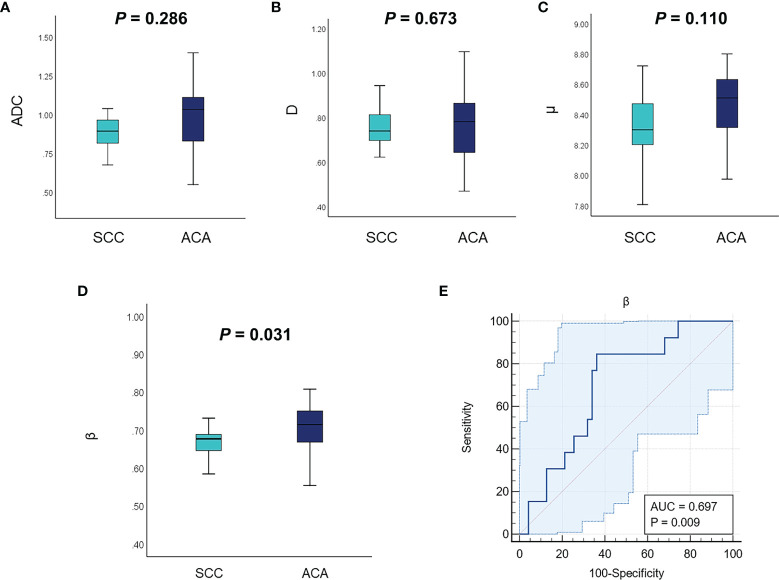
Comparison of the mean ADC **(A)**, *D* **(B)**, μ **(C)**, and β **(D)** between different tumor subtypes using the Mann–Whitney U test. **(E)** The ROC curve of using β for classification of SCC and ACA. ADC, apparent diffusion coefficient; ROC, receiver operating characteristic; SCC, cervical squamous cell carcinoma; ACA, cervical adenocarcinoma.

**Figure 1** shows a set of representative anatomic images and corresponding β, *D* and μ maps for a 70-year-old patient with SCC and a 56-year-old patient with ACA. The descriptive statistics of ADC and the FROC model parameters from each patient group are summarized in **Table 3**. As shown in **Figure 2**, no significant result was found for ADC, *D* and *μ* in differentiation SCC lesions from ACA lesions. However, β derived from the FROC model could differentiate cervical carcinoma subtypes (*P* = 0.031 <0.05) and the SCC lesions exhibited significantly lower β than that in the ACA lesions. For the ROC analysis, β proved to be the significant predictor with the best cut-off value 0.697 (AUC = 0.697, 95% confidence interval 0.565 to 0.809; sensitivity = 63.8%, specificity = 84.6%, accuracy = 68.3%).

**Table 3:** The descriptive statistics of ADC and FROC model parameters from different groups.

	ADC	β	*D*	μ
**SCC**	0.92 ± 0.17 (0.90, 0.36)	0.67 ± 0.05 (0.68, 0.18)	0.77 ± 0.12 (0.74, 0.32)	8.19 ± 0.80 (8.28, 0.91)
**ACA**	1.00 ± 0.27 (1.04, 0.85)	0.71 ± 0.08 (0.72, 0.26)	0.80 ± 0.20 (0.83, 0.63)	8.40 ± 0.31 (8.50, 0.82)
***P***	0.286	0.031	0.673	0.110
**Low-grade**	0.98 ± 0.19 (0.96, 0.49)	0.73 ± 0.06 (0.72, 0.18)	0.82 ± 0.13 (0.80, 0.28)	8.27 ± 0.93 (8.48, 1.19)
**High-grade**	0.90 ± 0.20 (0.86, 0.65)	0.68 ± 0.08 (0.68, 0.23)	0.74 ± 0.14 (0.72, 0.47)	8.26 ± 0.36 (8.27, 0.53)
***P***	0.022	0.009	0.004	0.015

Values are given as mean ± SD (median, range). β is unitless; ADC and D with unit (×10^−3^ mm^2^/s); μ with unit (μm).

P-values are statistical comparisons between different tumor subtypes and histological grades.

ADC, apparent diffusion coefficient; FROC, fractional order calculus; SCC, cervical squamous cell carcinoma; ACA, cervical adenocarcinoma.

### Comparative Analysis of ADC and the FROC Parameters in Cervical Carcinoma Grade

**Figure 3:**
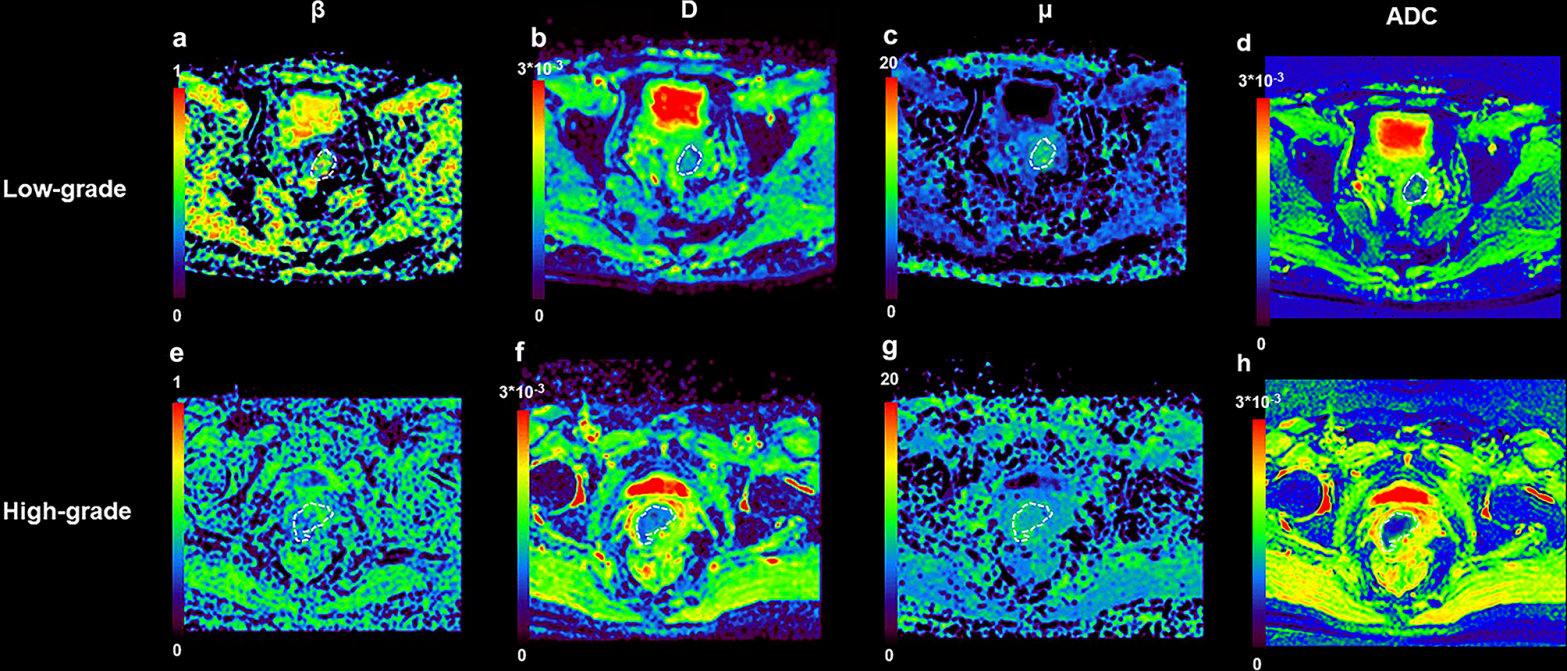
An example of low-grade tumor from a 53-year-old patient and high-grade from a 64-year-old patient. **(A)** β map for patient with low-grade tumor; **(B)** *D* map for patient with low-grade tumor; **(C)** μ map for patient with low-grade tumor; **(D)** ADC map for patient with low-grade tumor; **(E)** β map for patient with high-grade tumor; **(F)** *D* map for patient with high-grade tumor; **(G)** μ map for patient with high-grade tumor; **(H)** ADC map for patient with high-grade tumor.

**Figure 4:**
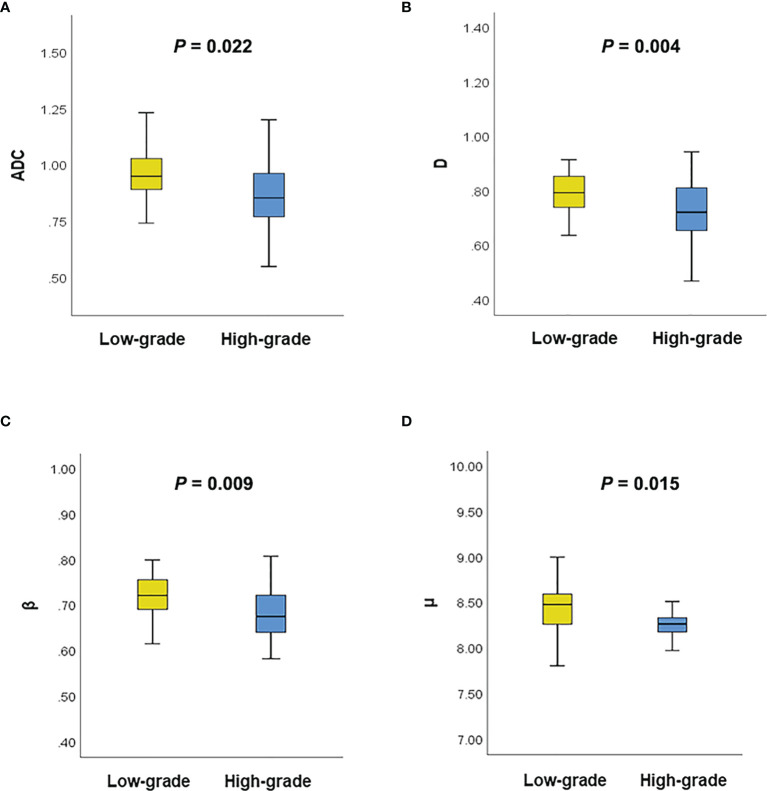
**(A)** Comparison of the ADC between different tumor grades using the Mann–Whitney U test; **(B)** Comparison of the *D* between different tumor grades using the Mann–Whitney U test; **(C)** Comparison of the β between different tumor grades using the Mann–Whitney U test; **(D)** Comparison of the μ between different tumor grades using the Mann–Whitney U test.

**Figure 5:**
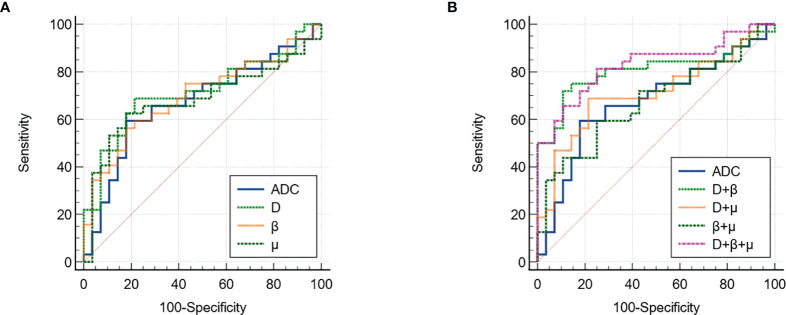
The ROC curves of using **(A)** individual and **(B)** different combinations of FROC parameters for differentiation of low-grade tumor and high-grade tumor. ROC, receiver operating characteristic; FROC, fractional order calculus.

**Figure 3** shows a set of β, *D*, *μ* and ADC maps for a 53-year-old patient with low-grade cervical carcinoma and a 64-year-old patient with high-grade one. As shown in **Figures 4A–D**, all the individual parameters, namely, ADC, β, *D* and *μ*, could differentiate low-grade cervical carcinoma from high-grade ones (*P* = 0.022, 0.009, 0.004, and 0.015, respectively). The high-grade lesions exhibited significantly lower ADC, β, *D* and *μ* than those in the low-grade lesions. **Figure 5A** and associated **Table 4** show the results from the ROC analysis. Among all the parameters, exhibited the best overall performance and outperformed ADC in AUC *D* (0.714 vs. 0.673), sensitivity (68.7% vs. 59.4%), and accuracy (73.3% vs. 70.0%). However, the differences between sensitivities, specificities, accuracies, and AUC values were not significant among all these individual parameters.

**Table 4:** The ROC analysis results of using ADC and individual FROC model parameters to differentiate low-grade tumor from high-grade tumor.

	ADC	β	*D*	μ
**Sensitivity**	59.4%	56.2%	68.7%	62.5%
**Specificity**	82.1%	82.1%	78.6%	82.1%
**Accuracy**	70.0%	68.3%	73.3%	71.7%
AUC*	0.673 (0.540–0.789)	0.696 (0.564–0.809)	0.714 (0.583–0.824)	0.683 (0.550–0.797)

ROC, receiver operating characteristic; ADC, apparent diffusion coefficient; FROC, fractional order calculus; AUC, are under the curve.

^*^Data in parenthesis are 95% confidence intervals.

The combinations of the FROC parameters further improved the differentiation of low-grade tumors from high-grade ones. All the combinations, namely, *D* + β, *D*+ *μ*, β + *μ*, and *D*+ β + *μ*, yielded significant differences (*P <*0.05). As shown in **Figure 5B** and associated **Table 5**, the combination of all the FROC parameters *D* + β + *μ* showed the best overall performance, producing the highest sensitivity (81.2%) and AUC (0.829). Significantly higher sensitivity (*P* = 0.016) and AUC (*P* = 0.043) were observed in the combination *D* + β + *μ* than in ADC. The combination of *D* and β showed the highest specificity (89.3%) and accuracy (80.0%). Though *D* + β had no significant differences from ADC in specificity (*P* = 0.500), its accuracy was significantly higher than ADC (*P* = 0.031).

**Table 5:** The ROC analysis results of using ADC and the combinations of the FROC model parameters to differentiate low-grade tumor from high-grade tumor.

	ADC	*D* + β	*D* + μ	β + μ	*D* + β+ μ
**Sensitivity**	59.4%	71.9%	68.7%	59.4%	81.2%
**Specificity**	82.1%	89.3%	78.6%	75.0%	75.0%
**Accuracy**	70.0%	80.0%	73.3%	66.7%	78.3%
AUC*	0.673 (0.540–0.789)	0.807 (0.685–0.897)	0.711 (0.579–0.821)	0.672 (0.539–0.788)	0.829 (0.710–0.914)

ROC, receiver operating characteristic; ADC, apparent diffusion coefficient; FROC, fractional order calculus; AUC, are under the curve.

^*^Data in parenthesis are 95% confidence intervals.

## Discussion

In this study, the feasibility of using the FROC model to classify cervical carcinoma subtypes and histological grades was investigated. The results demonstrated that only β derived from the FROC model could differentiate SCC from ACA. The combination of all the FROC parameters provided the highest overall performance in identifying high-grade tumors from low-grade ones, demonstrating 0.829 AUC, 81.2% sensitivity, 75.0% specificity, and 78.3% accuracy. Significantly higher sensitivity (*P* = 0.016) and AUC (*P* = 0.043) were observed in the combination *D*+ β + *μ* than in ADC.

Prior studies suggested that different cervical carcinoma subtypes had different treatment outcome and prognosis. Hopkins et al. demonstrated that ACA had a worse 5-year overall survival rate of 15–30% compared to SCC in all stages (21). Katanyoo et al. reported that ACA had more radio resistance than SCC. ACA in locally advanced cervical cancer had poorer response rate from radiation therapy and concurrent chemoradiation and also took a longer time to achieve complete response than SCC (22). Consequently, differentiation between SCC and ACA in an early time is critical for treatment decision and patient management. With its sensitivity to tissue structural and functional alterations, DWI has been routinely used in conjunction with T2-weighted imaging for tumor detection. In cervical cancer, the lower ADC in tumors compared with non-tumor epithelium provides excellent tumor-to-normal-tissue contrast (23, 24). However, the potential of ADC values to differentiate the cervical carcinoma subtypes remains controversial. Some studies reported that the ADC values of SCC were significantly lower than those of ACA (6, 25), whereas Winfield et al. showed that ADC values could not differentiate SCC from ACA (26). In the present study, no significant difference was found for ADC between the tumor subtypes, while β was significantly lower for the SCC lesions than ACA ones. The diffusion heterogeneity parameter in the FROC model has been increasingly focused in recent literatures. Unlike all previous studies on cervical carcinoma using conventional DWI model, the FROC model is able to probe the tissue microstructural information with high b-values and increase the diagnostic performance to some extent. Prior research suggested that *D* reflected intrinsic diffusivity, μ was related to mean free length of diffusion, and β had a significant negative correlation with increased intravoxel tissue heterogeneity (11, 17, 27, 28). This study suggested a significant increase of tissue heterogeneity of SCC compared to ACA, which agreed with the findings in previous conventional DWI and diffusion kurtosis imaging (DKI) studies in cervical carcinoma. For instance, Wang et al. reported that mean diffusivity (MD) in SCC was significantly lower than that in ACA, and the lower MD in cervical carcinoma was likely related to the restriction of free water diffusion in more cellular packed tumor environment (29).

Aside from differentiation of the subtypes, cervical cancer grading also relies on invasive biopsy, which introduces the location bias. Therefore, developing non-invasive imaging biomarkers to assist tumor grading on the basis of MR images holds clinical significance. Previous studies reported that the FROC parameters could effectively distinguish tumor grades in glioma (30), pediatric brain tumor (28), and prostate cancer (16). In this study, the high-grade lesions exhibited significantly lower ADC, β, *D*, and *μ* than those in the low-grade ones and *D* derived from the FROC model provided the best individual parameter performance in grading the tumors compared with the other parameters. Similarly, prior studies found that ADC was negatively correlated with tumor grades of cervical carcinoma, indicating the aggressiveness of cervical carcinoma (31, 32). Related literatures suggested that the high-grade tumor resulted in increased cellular density, enlarged nuclei, and higher nuclear-to-cytoplasmic ratio with a more heterogeneous microenvironment (32, 33). However, the considerable overlap of ADC values among different tumor grades may limit the value of conventional DWI in diagnosis (8, 9). Furthermore, the high-grade tumors can have a higher degree of tissue heterogeneity, a characteristic that may not be adequately captured in a simple ADC value obtained from a mono-exponential diffusion model (34, 35).

It should be noted that β was found to be the only parameter which showed significance among the different cervical cancer subtypes; however, it is the one with the lowest diagnostic accuracy in differentiation among the low- and high-grade cervical carcinoma. These results may be attributed to the complex network of tumor microenvironment. The mechanism of differentiation of tumor subtype probably different from the differentiation of tumor grade. Results were also mixed in previous DWI-related studies. For instance, according to Wang et al., MD derived from DKI model and conventional ADC were significantly lower in squamous cell carcinoma than in adenocarcinoma, while no difference was observed in different tumor grade (29). Besides, Winfield showed that α from the stretched exponential model, K from the kurtosis model, and f and D* from the bi-exponential model were significantly different between types of tumor, while ADC from the mono-exponential model, DDC from the stretched exponential model, DK from the kurtosis model, Ds’ from the statistical model and D from the bi-exponential model were significantly different between tumor grades (26).

Compared to the conventional mono-exponential diffusion model that is limited to the single parameter ADC, the FROC model has the preponderance of the ability of combining multiple parameters, which greatly improved the diagnostic performance. This combination approach suggests that multiple tissue properties, namely, cellularity, microstructures, and heterogeneity, can complement each other and contribute to the diagnosis and prognosis of tumors simultaneously. In the present study, the combination of all the FROC parameters showed the best overall performance in grading the cervical carcinoma, producing the highest sensitivity (81.2%) and AUC (0.829). The *D* + β showed the highest specificity (89.3%) and accuracy (80.0%). These multivariate analysis results further suggested that a noninvasive DWI-based classifier which reflected tumor grade of cervical carcinoma could be developed.

This study has several limitations. First, this is a single-site study with relatively small sample size and only 13 cases of ACA were included, which could reduce the accuracy of results. Further study with more patients would be needed. Second, due to the limited cases of ACA, we did not evaluate the tumor grade separately based on different tumor subtypes, which may lead to the potential bias and decrease the specificity of results. Third, rare histological subtypes, especially adenosquamous carcinoma, were excluded from this study. However, these rare tumor subtypes tend to have a poorer prognosis and remain difficulty in imaging diagnosis (36). Further investigation with more such cases should be conducted to validate this preliminary result and provide more robust support for clinical decision making. Finally, in order to take full advantage of the FROC model, b-values must be sufficiently high to accentuate non-Gaussian diffusion behaviors. Nevertheless, in this study, the maximal b-value was limited to 2,000 s/mm^2^, and the higher b-values were more sparsely sampled than the lower b-values due to the efficiency considerations. The effect of the selection of b-value on the FROC model needs to be investigated in future study.

In conclusion, this study demonstrated the feasibility of using non-Gaussian diffusion FROC model to differentiate the tumor subtypes and histological grades of cervical carcinoma. In particular, the FROC model provided a set of novel diffusion parameters and the combination of these parameters contributed to the best diagnostic performance in differentiation between low-grade and high-grade cervical carcinoma. This advanced diffusion approach could be a noninvasive and *in vivo* diagnostic technique in cervical carcinoma, which is conducive to treatment decision and patient management.

## Data Availability Statement

The datasets generated for this study are available on request to the corresponding authors.

## Ethics Statement

The studies involving human participants were reviewed and approved by The Fourth Hospital of Hebei Medical University. The patients/participants provided their written informed consent to participate in this study.

## Author Contributions

XS: Writing—original draft, Investigation. LA: Writing—original draft, Formal analysis. HL: Visualization, Data curation. HF: Resources, Data curation. LZ: Software, Validation. YD: Methodology. JZ: Writing—review & editing, Project administration. BY: Writing—review & editing, Supervision. All authors listed have made a substantial, direct, and intellectual contribution to the work and approved it for publication.

## Conflict of Interest

Authors LZ and YD were employed by United Imaging Healthcare.

The remaining authors declare that the research was conducted in the absence of any commercial or financial relationships that could be construed as a potential conflict of interest.

## Publisher’s Note

All claims expressed in this article are solely those of the authors and do not necessarily represent those of their affiliated organizations, or those of the publisher, the editors and the reviewers. Any product that may be evaluated in this article, or claim that may be made by its manufacturer, is not guaranteed or endorsed by the publisher.
